# Tongue feature dataset construction and real-time detection

**DOI:** 10.1371/journal.pone.0296070

**Published:** 2024-03-07

**Authors:** Wen-Hsien Chang, Chih-Chieh Chen, Han-Kuei Wu, Po-Chi Hsu, Lun-Chien Lo, Hsueh-Ting Chu, Hen-Hong Chang

**Affiliations:** 1 Graduate Institute of Chinese Medicine, School of Chinese Medicine, College of Chinese Medicine, China Medical University, Taichung, Taiwan, Republic of China; 2 Center for Artificial Intelligence in Medicine, Chang Gung Memorial Hospital, Taoyuan, Taiwan, Republic of China; 3 School of Post-Baccalaureate Chinese Medicine, College of Chinese Medicine, China Medical University, Taichung, Taiwan, Republic of China; 4 Department of Traditional Chinese Medicine, Kuang Tien General Hospital, Taichung, Taiwan, Republic of China; 5 School of Chinese Medicine, College of Chinese Medicine, China Medical University, Taichung, Taiwan, Republic of China; 6 Department of Chinese Medicine, China Medical University Hospital, Taichung, Taiwan, Republic of China; 7 Department of Computer Science and Information Engineering, College of Computer Science, Asia University, Taichung, Taiwan, Republic of China; 8 Graduate Institute of Integrated Medicine, College of Chinese Medicine, and Chinese Medicine Research Center, China Medical University, Taichung, Taiwan, Republic of China; Vellore Institute of Technology: VIT University, INDIA

## Abstract

**Background:**

Tongue diagnosis in traditional Chinese medicine (TCM) provides clinically important, objective evidence from direct observation of specific features that assist with diagnosis. However, the current interpretation of tongue features requires a significant amount of manpower and time. TCM physicians may have different interpretations of features displayed by the same tongue. An automated interpretation system that interprets tongue features would expedite the interpretation process and yield more consistent results.

**Materials and methods:**

This study applied deep learning visualization to tongue diagnosis. After collecting tongue images and corresponding interpretation reports by TCM physicians in a single teaching hospital, various tongue features such as fissures, tooth marks, and different types of coatings were annotated manually with rectangles. These annotated data and images were used to train a deep learning object detection model. Upon completion of training, the position of each tongue feature was dynamically marked.

**Results:**

A large high-quality manually annotated tongue feature dataset was constructed and analyzed. A detection model was trained with average precision (AP) 47.67%, 58.94%, 71.25% and 59.78% for fissures, tooth marks, thick and yellow coatings, respectively. At over 40 frames per second on a NVIDIA GeForce GTX 1060, the model was capable of detecting tongue features from any viewpoint in real time.

**Conclusions/Significance:**

This study constructed a tongue feature dataset and trained a deep learning object detection model to locate tongue features in real time. The model provided interpretability and intuitiveness that are often lacking in general neural network models and implies good feasibility for clinical application.

## Introduction

Traditional Chinese medicine (TCM) physicians learn about the status of internal and external organs, meridians, and blood-Qi circulation in the human body, infer physiological and pathological changes, and select appropriate treatments through the application of four methods of diagnosis: inspection, listening and smelling examinations, inquiry, and palpation. Tongue examination is part of the inspection diagnosis, since the condition of the tongue is often highly correlated with a patient’s health status and disease course. Inspecting specific tongue features provides TCM physicians with clinically important, objective evidence that assists with diagnosis, whereas patients’ narratives can contribute to diagnostic errors. Tongue diagnosis is therefore widely used by TCM physicians.

Importantly, the determination of tongue features can be subjectively affected by observation, with different TCM physicians disagreeing about the interpretation of features on the same tongue, leading to different study conclusions [1–3]. Moreover, experienced TCM physicians typically identify tongue features that are overlooked by nonclinical personnel [1–3]. In addition, in the current medical process, to record and interpret a patient’s tongue features, the patient is guided to an examination room first. Then, an assistant captures only “one” tongue image in a well-prepared photographic environment. The interpretation report is completed by a TCM physician after a few days. The reports are in plain text format and mainly specify which tongue features exist, without precise positioning information. The development of an automated tongue feature detection system would expedite the interpretation process and make it possible to obtain more consistent results and reduce human errors. Moreover, junior doctors and medical students could learn tongue diagnosis more efficiently.

In the past few decades, automated interpretation of the tongue has been performed through conventional feature extraction algorithms and statistical methods. For example, conventional image processing techniques have been used to detect tongue features with their corresponding areas [4–6]. However, those studies lack detailed assessment methods and results [4, 5]. In recent years, artificial intelligence (AI) has been actively applied to medical technology, and significant progress with deep learning image processing has eliminated the need for manual extraction of image features [7, 8]. Furthermore, transfer learning has allowed big datasets to pretrain a deep learning model that can often be easily used to interpret image categories of other, different big datasets. For example, Iqbal *et al*. applied transfer learning to detect the synovial fluid of human knee joint [9]. Several studies also applied Gradient-weighted Class Activation Mapping or other visualization techniques to roughly locate tongue features [10–12]. Only two applied deep learning object detection techniques to mark tongue features. Weng *et al*. detected fissures and tooth marks with rectangles, but the marking was coarse and details of the dataset construction were not described [13]. Zhang *et al*. detected several features but the performance was not clear [53]. This study built a manually annotated dataset for several tongue features in TCM and applied the well-known deep learning object detection model "You Only Look Once v4-tiny (YOLOv4-tiny)" [14]. The model marks tongue features with rectangles, so is not limited to simply determining the existence of a tongue feature. Users can clearly see the locations of tongue features.

**Fig 1:**
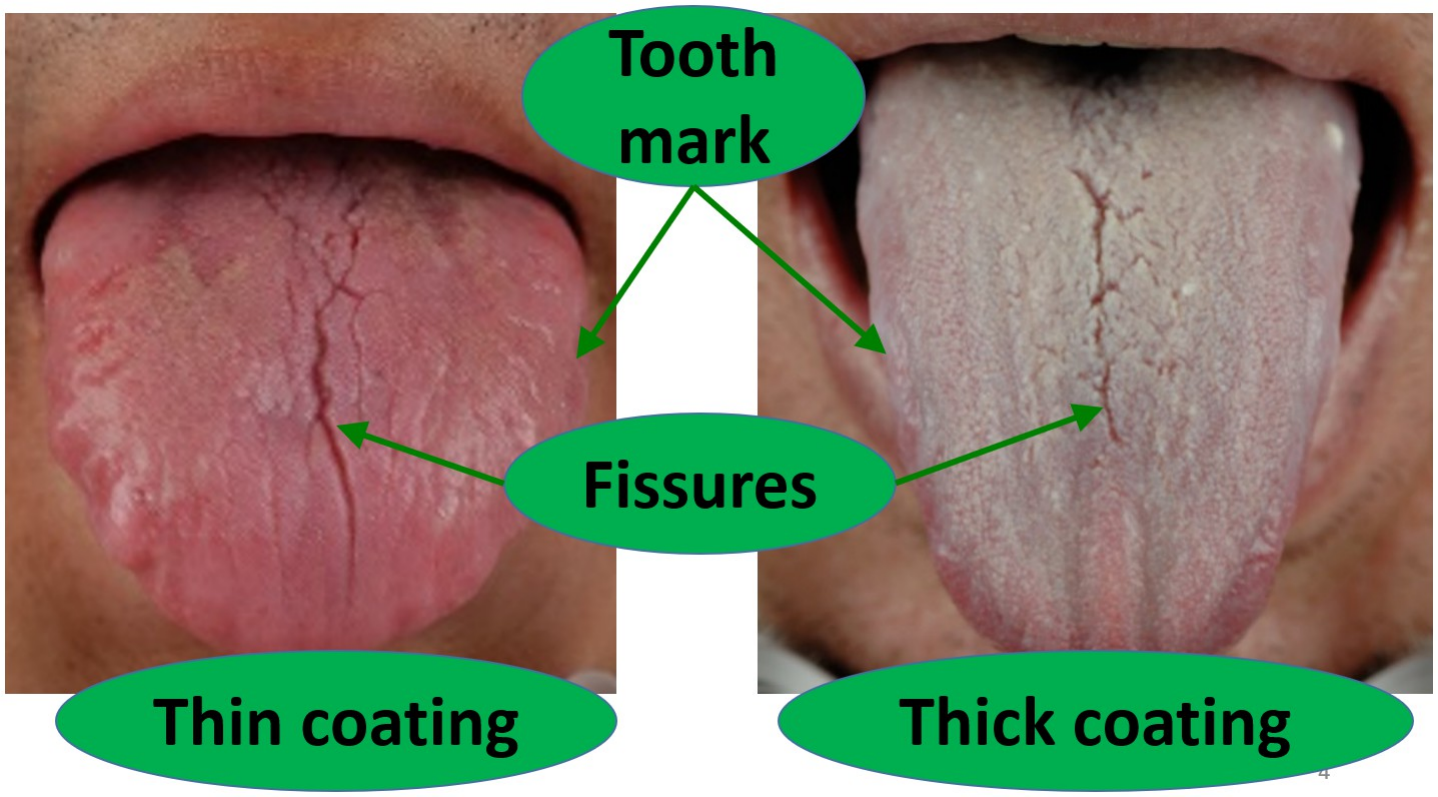
Examples of tongue features. Both tongues contain tooth marks on the edges and fissures in the middle. The tongue on the left has a thin coating, while the one on the right has a thick coating.

Many tongue features are clinically examined in TCM, including tongue fissures, tooth marks, and thin and thick coatings, as shown in Fig 1. The clinical significance of these features can be interpreted from the perspectives of TCM and modern medicine.

### Tongue fissures

Some researchers believe that fissured tongues are hereditary [15] and positively correlated with age and male sex [16]; fissures are very rare in children aged less than 10 years [17]. A burning sensation in the mouth is more relevant to tongue fissures [18, 19] and similar to the TCM concept that fissures arise from excessive heat or inadequate body fluid [20]. Generally, a fissured tongue does not directly indicate a specific disease. For instance, the reported incidence of tongue fissures ranges widely (from 20%–95%) in patients with Down syndrome in three different studies [21–23] and also in patients with psoriasis (from 4%–66%) [24–26]. Not only do the incidence rates of fissured tongue vary widely in specific diseases, but also, it remains to be clarified as to whether tongue fissures are simply a normal phenomenon of age from adolescence upwards, and when tongue fissures have pathological significance. Tongue fissures lack a consistently high standard of interpretation.

### Tooth marks

Tooth marks can be caused by an excessive size of the tongue [27]. Some studies have found that tooth marks are associated with three obesity-related disorders; obstructive sleep apnea [28], nocturnal intermittent hypoxia, and snoring [29], each of which disturb sleep and lead to mental fatigue. In TCM diagnostics, obesity has the symptom of “dampness” (resulting from fat in the body), while a deficiency in qi (vital energy) is marked by fatigue [20]. Tooth marks are important indicators for these contributing factors to illness in TCM theory.

### Thick coating

Many recent studies have explored the phenomenon of thick coatings on the tongue. A thick coating is positively correlated with bad breath [30] and *Helicobacter pylori* infection [31]. In another study, in which 459 patients with dysphagia sought medical attention, the thickness of the tongue coating was negatively correlated with food intake [32]. The findings in these three studies are similar to the phenomenon of dampness in the middle burner or food masses (indigestion) in TCM [20].

### Yellow coating

Smoking, poor oral hygiene, food and medications can result in tongue coating discoloration [33, 34]. Yellow coatings on tongues may be related to specific diseases. In a Japanese study involving 969 individuals aged 30–79 years, a yellow coating on the tongue was associated with diabetes [34], while Chinese research has reported finding that the extent of yellow coating (light yellow, or yellow) was associated with different types of eczema (subacute, acute, or chronic) [35]. In another study, around 75% of patients with chronic gastritis had a yellow coating on the tongue and almost all of those infected with *H*. *pylori* had a yellow coating [31]. A yellow coating is also associated with the presence of *Bacillus* on the tongue [36]. Yellow coatings on tongues in individuals with diabetes, eczema and bacterial infections are similar to the phenomena associated with dampness-heat (such as chronic inflammation or infection) in TCM theory [20].

## Materials and methods

### Ethical statement

This research was reviewed and approved by the institutional review board of China Medical University Hospital (registration number CMUH107-REC2-146). Data were obtained from China Medical University Hospital in April 2020. The data were analyzed anonymously. Informed consents were obtained from all participants. Participants provided consents when they first provided their data.

### Overview of the dataset and training process

**Fig 2:**
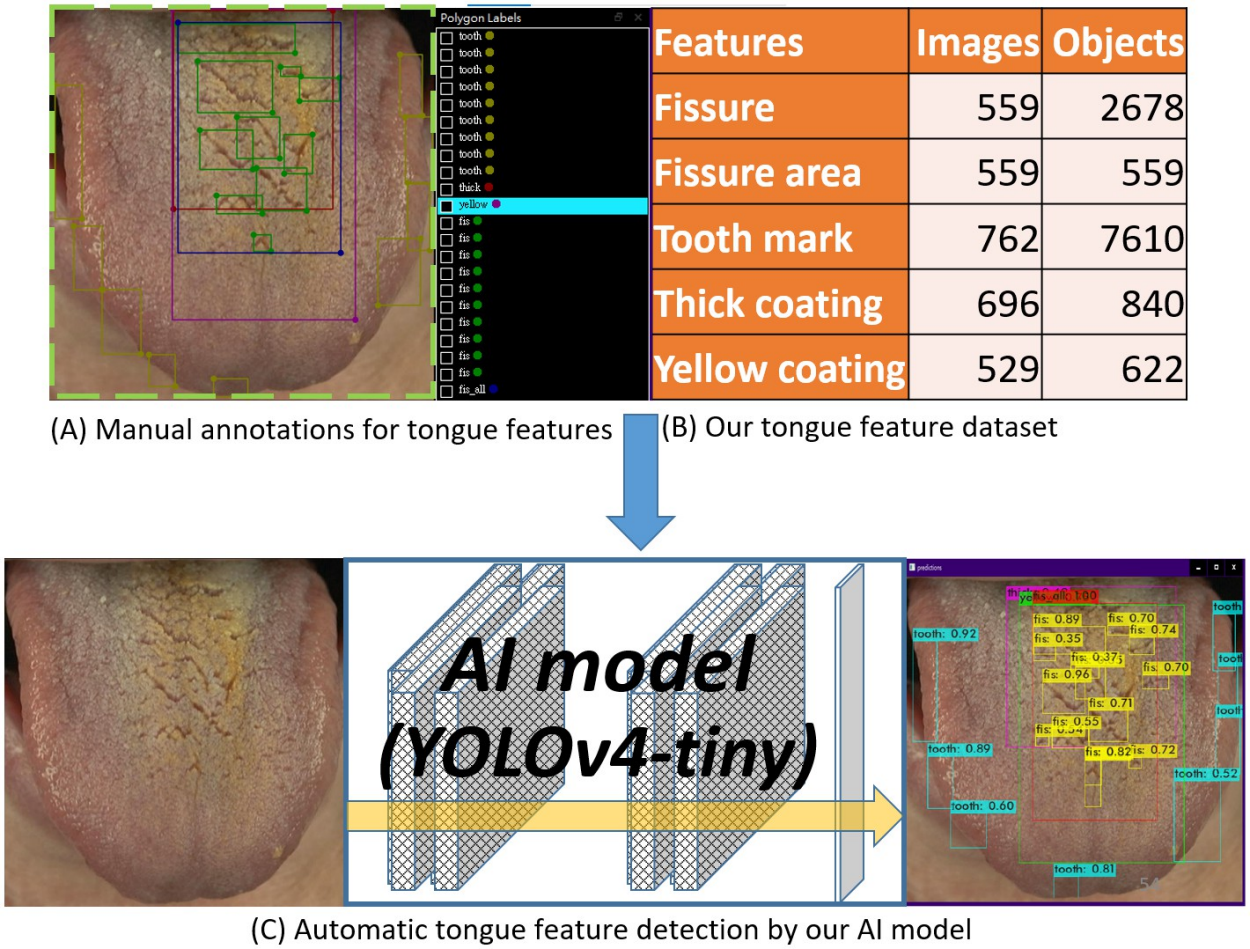
Overview of the study.

The overview of this study is illustrated in Fig 2.

**Fig 3:**
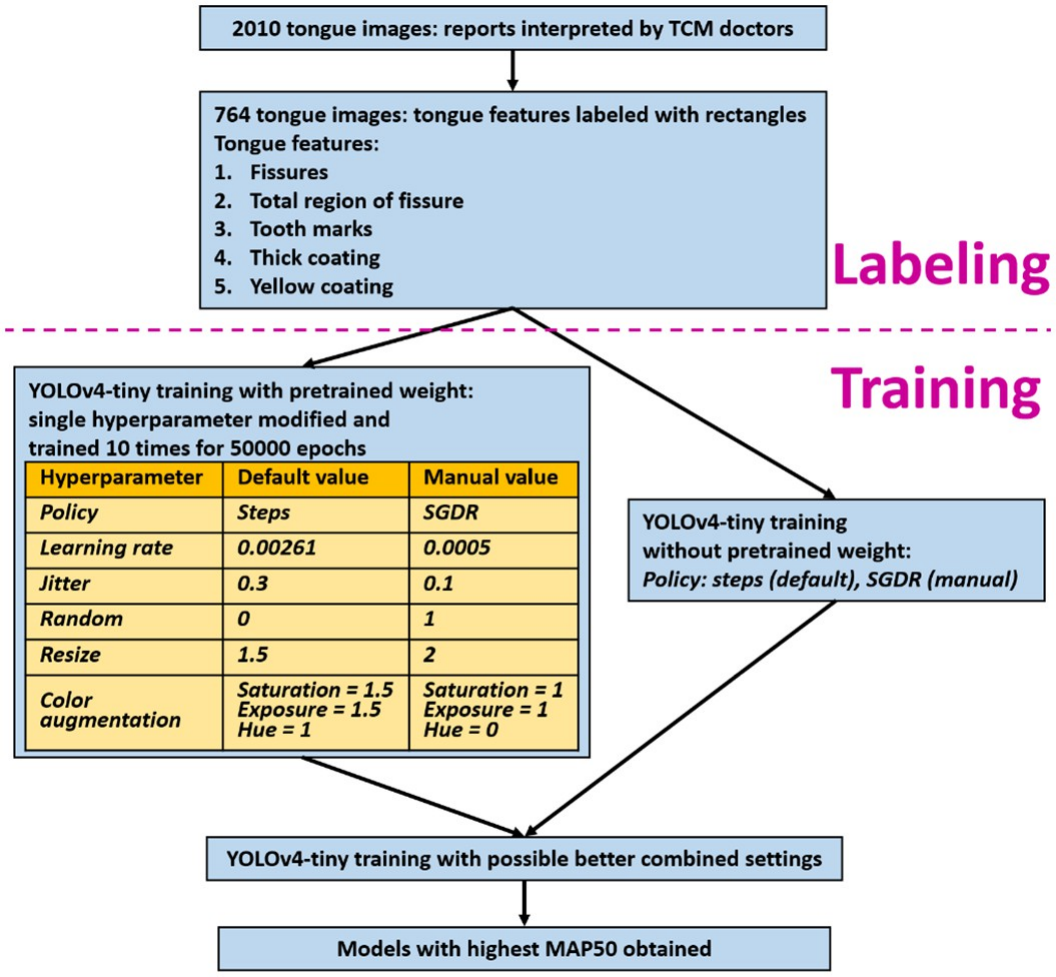
The development of tongue feature dataset and training process of a deep learning model.

The construction of the tongue feature dataset and training process of the deep learning model is illustrated in Fig 3 and described in detail below.

### Tongue feature annotation process and analysis

Currently, no publicly available manually annotated dataset exists for TCM tongue features. Deep learning object detection techniques require the preparation of a huge number of annotated images with specific features. In this study, tongue images recorded over several years in the Department of Chinese Medicine of China Medical University Hospital were used to construct a dataset of tongue features. The tongue diagnosis data cover the period from January 2008 through March 2020 and include a total of 2,010 images and corresponding interpretation reports issued by TCM physicians. The interpretation reports are in plain text format and mainly specify which tongue features exist, without precise positioning information. In the environment for capturing tongue images, the digital camera, ring light, and chin rest were stably set up and covered with a focusing cloth. Participants were guided to stabilize their chins on the chin rest and protrude their tongues to the appropriate position for capturing. For 764 images in this dataset, tongues and tongue features such as tongue fissures, tooth marks, thick coatings and yellow coatings have been manually annotated with minimal rectangles using an assisted annotation tool LabelMe [37]. These 764 images belong to 652 different tongues and were obtained in a darkroom on different dates. More images will be annotated in the future.

**Fig 4:**
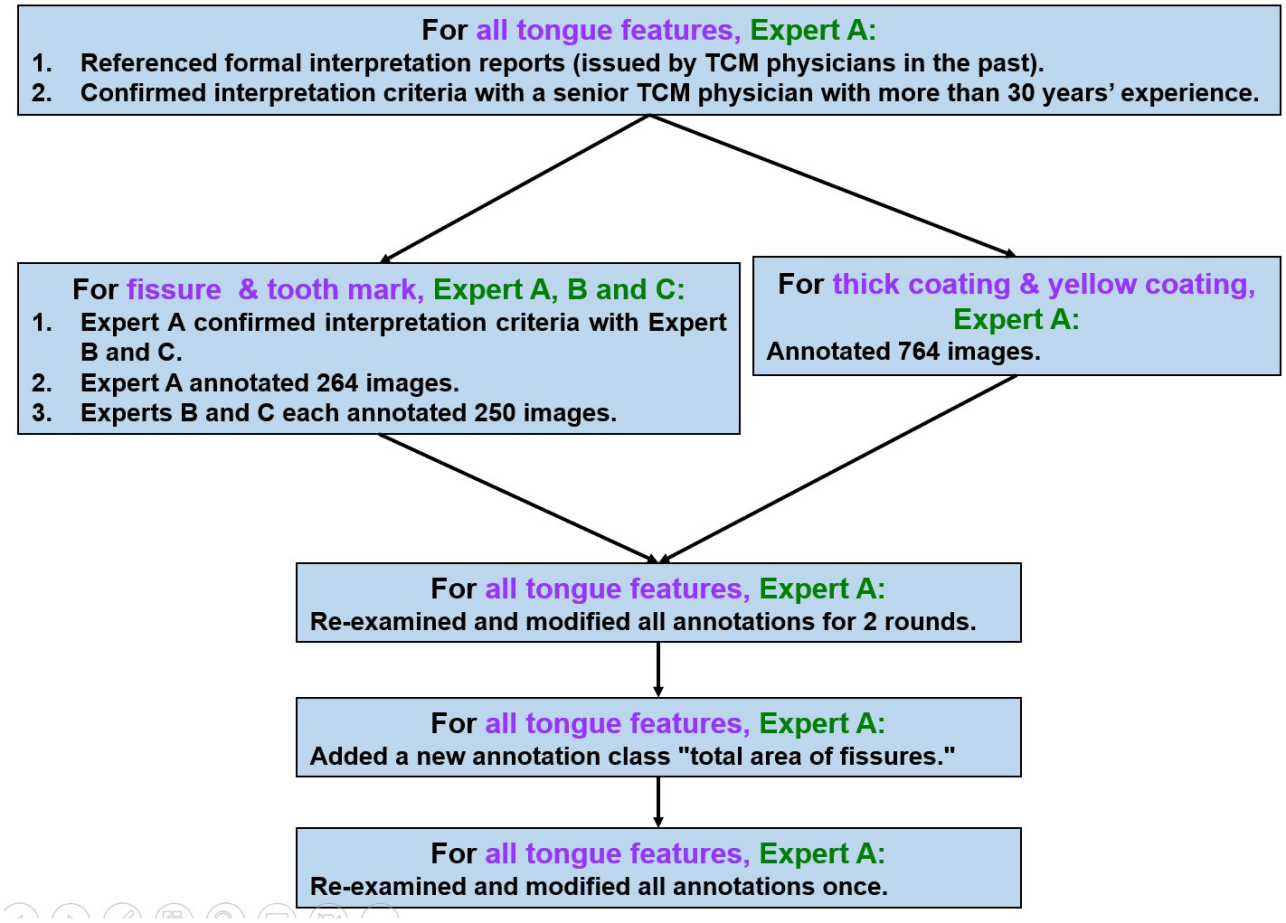
The tongue feature annotation process.

The tongue feature annotation process is illustrated as Fig 4. TCM Expert A referenced formal interpretation reports (issued by TCM physicians in the past) and confirmed the interpretation criteria with a senior TCM physician with more than 30 years’ experience, before commencing the first round of annotation. Tongues, thick coatings and yellow coatings were annotated by Expert A. With regard to tongue fissures and tooth marks, because of higher interobserver agreement for these features [1], TCM Experts B and C were included to assist with annotation. Expert A annotated 264 images, while Experts B and C each annotated 250 images. In order to ensure consistency among all three Experts, 15 images were firstly annotated by Experts B and C, then modified one by one by Expert A. Subsequently, the same process was followed for another 15 images, then finally for another 20 images. After a higher consistency was achieved, Experts B and C each separately annotated an additional 200 images.

After this first round of annotation, Expert A re-examined and modified in detail all annotations in all images for two rounds. Due to the large variation of fissure sizes and occurrences of tens of fissures on a single tongue, a new annotation class ("total area of fissures") was created, whereby a single minimal rectangle enclosed all fissures in each tongue. This new annotation can crop the total area of fissures and yield a higher-resolution image that includes all fissures, enabling higher accuracy of fissure detection. Finally, Expert A re-examined and modified in detail all annotations once more. In summary, one round for initial annotation and three rounds for detailed modifications were performed. Fig 2A illustrates an example (from the set of 764 annotations) of how all tongue features were annotated to prepare the dataset.

### AI training

The above annotated tongue feature dataset was used to train the deep learning object detection model YOLOv4-tiny, the “tiny” version of the widely used object detection model YOLOv4.

The YOLOv4-tiny source code used in this study was obtained from https://github.com/AlexeyAB/darknet; the same website also provided pretrained weights for the publicly available MS COCO (Microsoft COCO: Common Objects in Context) dataset [38]. Following conventional training policy, 70% of the dataset was used for training and 30% for testing. An average precision with an intersection over union greater than or equal to 50% (AP50) was used as the evaluation metric. AP50 is a commonly used metric in the evaluation of performance of an object detection model and has been defined in detail in previous research [39]. In order to maximize the AP50 value, many of the model’s default training settings were modified, as described in the following text.

Using pretrained weights to perform transfer learning onto new, small datasets usually yields quite good effects quickly. Due to the large number of objects in this study dataset, the effects of training with and without pretrained weights were compared.

1. Color augmentation: the use of color augmentation prevents the model from overfitting the data from the training set, by adjusting the image saturation, exposure and hue. Thus, the model is better adapted to the variability of real-world tongue image coloring during tongue feature detection, although the interpretation of some features such as a thick coating and a yellow coating can be affected by changes in colors. Therefore, the effect of training with and without color augmentation for each tongue feature were compared.
2. Aspect ratios and image resizing: the differentiation ability of the model was increased by changing the aspect ratios and scales of images, to increase the variability by incorporating different aspect ratios and scale of tongue features. Three related parameters were involved in the model’s training settings:
  1. Jitter: this parameter denotes the degree of change in the aspect ratio of an image. Jitter changes the aspect ratio from (1–2*jitter) to (1 + 2*jitter) in the last layer of the model. Using the model’s default value of *0*.*3*, the study also tested the value of *0*.*1* (to decrease the degree of change in the aspect ratio of images) to compare the difference.
  2. Random: this parameter increases the degree of change in the network scale. Enabling (*random = 1*) randomly resizes the network size from *1/1*.*4* to *1*.*4* in the last layer of the model at every 10 epochs. As the default setting (*random = 0*) disables this technique, outcomes from both the default setting and the enabled setting (*random = 1*) were compared.
  3. Resize: this parameter denotes the degree of change in resized images. The scale of images is changed from *1/resize* to *resize* before training, using a default value of *1*.*5*. In this study, the difference with *2* (to increase the degree of change in image scales) was tested.
3. Learning rate selection: the best possible value for the learning rate is usually inferred through multiple experiments, or by using adaptive learning rate policies such as SGDR (Stochastic Gradient Descent with warm Restarts) [40–42]. The default initial learning rate is *0*.*00261* and changes to one-tenth of the origin (*0*.*000261*) when the training process achieves 80%, then changes to 1% of the origin (*0*.*0000261*) when the training process achieves 90%. SGDR and another initial learning rate of 0.005 were tested to compare differences in training results.

Each setting was used for 10 rounds (each containing 50,000 epochs) when training the model, in response to the results of several experiments demonstrating that the best result usually occurs before 50,000 epochs. Because different training rounds sharing the same setting may yield slightly different AP50 values, the best AP50 and the average AP50 values from 10 rounds were obtained to achieve a more objective view compared with single-round training. The combined values from these improved settings were used to train the model again, to achieve a model with the highest AP50 value.

## Results and discussion

The characteristics of the tongue feature dataset constructed in this study and the training results of the object detection model for the dataset are described separately in the following sections.

### Tongue feature dataset characteristics

**Fig 5:**
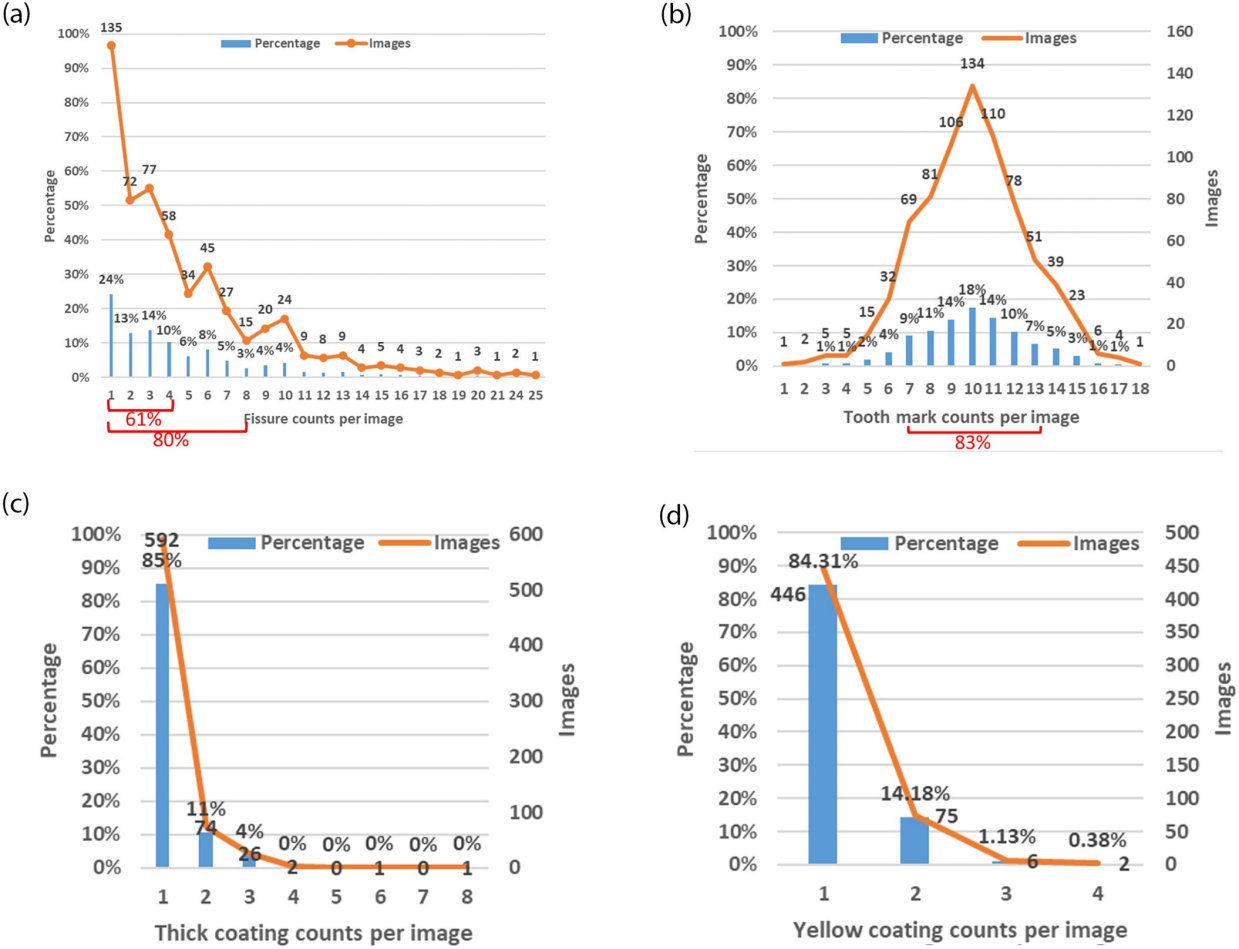
Distributions of tongue feature counts. (A) Distribution of fissure counts, (B) Distribution of tooth mark counts, (C) Distribution of thick coating counts, (D) Distribution of yellow coating counts.

The numbers of images containing each tongue feature and numbers of feature objects are illustrated in Table 1. Each feature object is annotated by a minimal rectangle, so for each tongue feature there may be several feature objects. The distributions of objects counted on each tongue depicted in Table 1 are illustrated in Fig 5.

**Table 1:** Numbers of images and numbers of feature objects in the dataset.

	Fissure	Total area of fissures	Tooth mark	Thick coating	Yellow coating
Numbers of images	559	559	762	696	529
Numbers of objects	2,678	559	7,610	840	622

### Fissures

**Fig 6:**
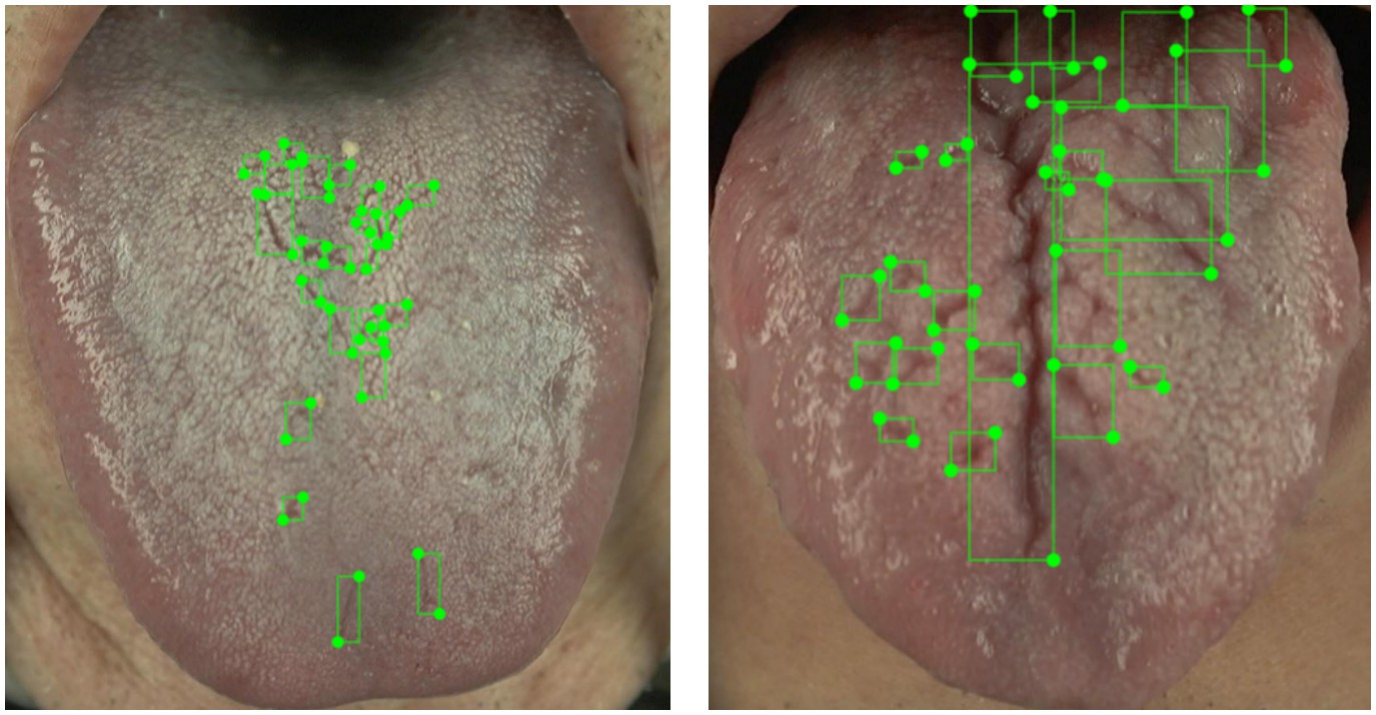
Tongues with multiple fissures.

**Fig 7:**
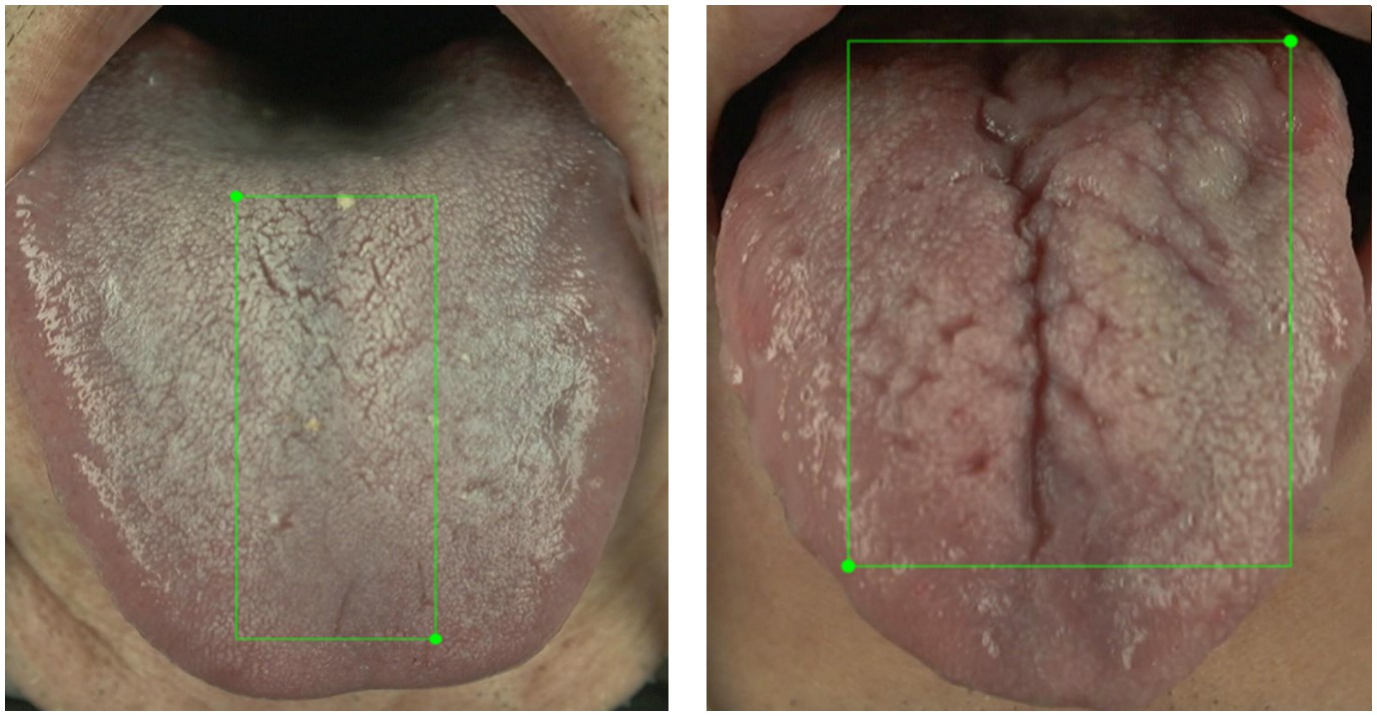
Total areas of tongue fissures.

A total of 73% of the images contain fissures; as illustrated in Fig 5A, around two-thirds (61%) of these images contain fewer than 5 fissures. Ambiguities exist in manual annotation. For example, Fig 6 depicts manually annotated tongues with more than 20 fissures, with crisscrossing of fissures that complicate the annotation, although their presence adds diversity to the dataset and they are therefore valuable contributors. Importantly, 80% of all images contain fewer than 8 fissures, which therefore ensures that during AI model training, the manual annotations still provide good consistency; a small number of ambiguous fissure patterns has little effect upon AI model training. When combined with annotations regarding the "total area of tongue fissures" as shown in Fig 7, the information is more than sufficient for clinical applications. From the TCM clinical point of view, the focus is on the presence or absence of fissures and the size of the fissure area, so the manual annotations should be sufficient for clinical use.

### Tooth marks

**Fig 8:**
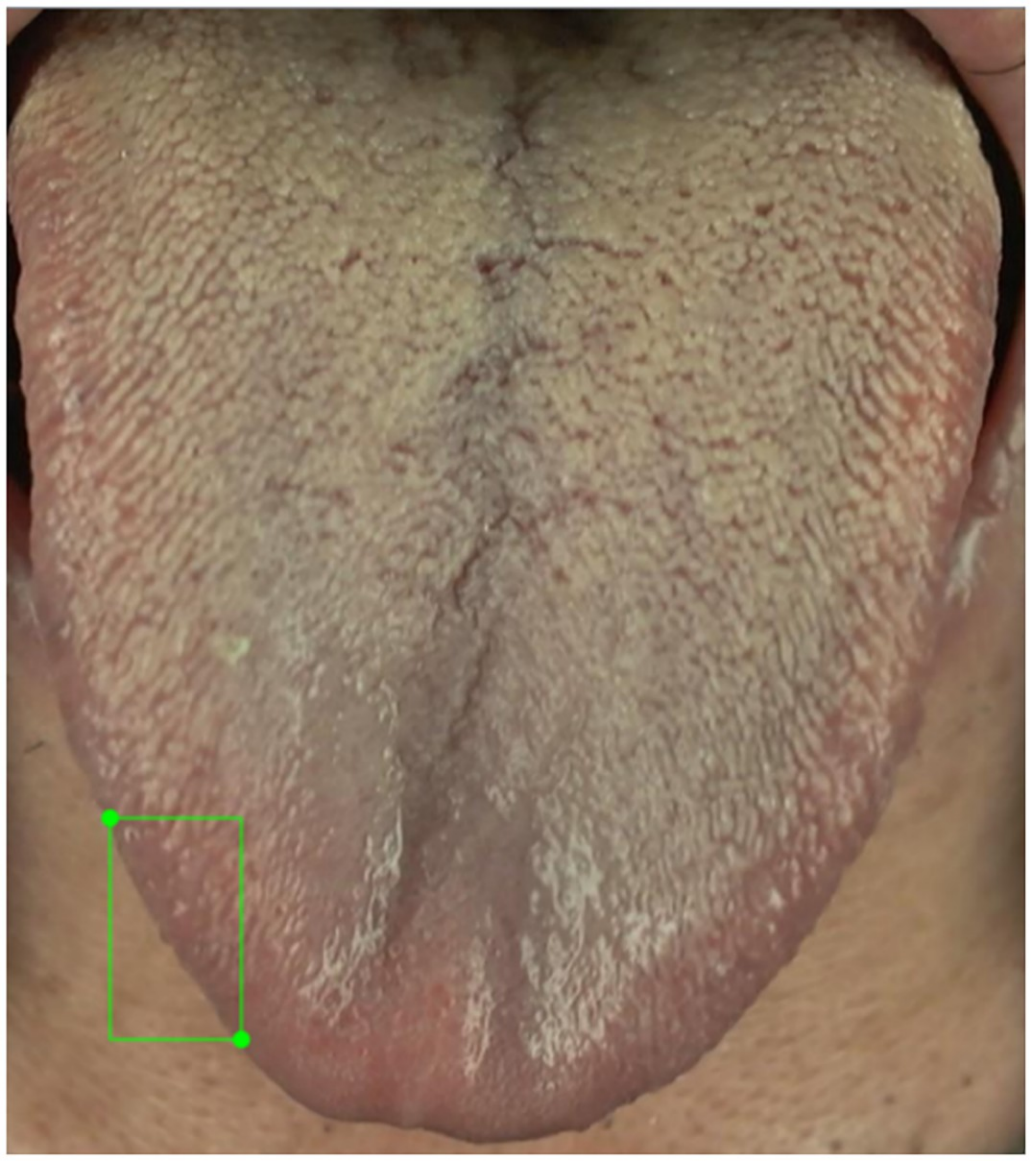
An indistinct tooth mark with darker color.

Almost all images contain tooth marks and 83% of the images contain between 7 and 13 tooth marks, as illustrated in Fig 5B. The depressions of some tooth marks are not obvious on the 2-dimensional plane image from a single viewpoint of a tongue, but appear only with a darker color, as shown in Fig 8. Manual annotation of indistinct tooth marks is difficult, because repeated inspections are required to minimize the degree of omission.

### Thick coatings

**Fig 9:**
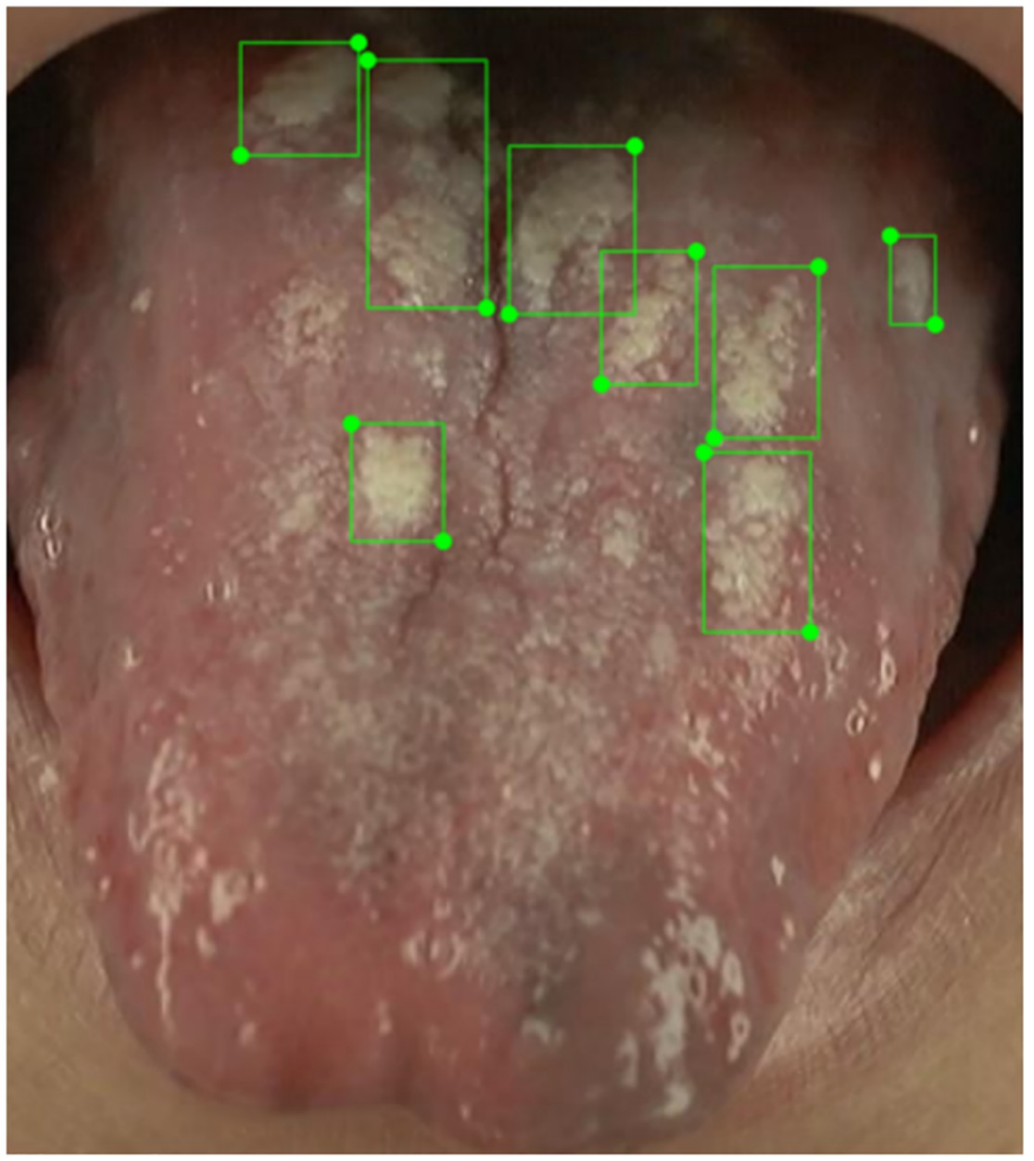
Tongues with multiple thick coating areas.

Most (91%) of the tongue images contain a thick coating. One tongue may contain multiple thick coating areas due to an uneven distribution of the tongue coating, as shown in Fig 9. Of the 696 images of tongues with thick coatings, most (85%) contain only one area of thick coating, as illustrated in Fig 5C.

### Yellow coatings

**Fig 10:**
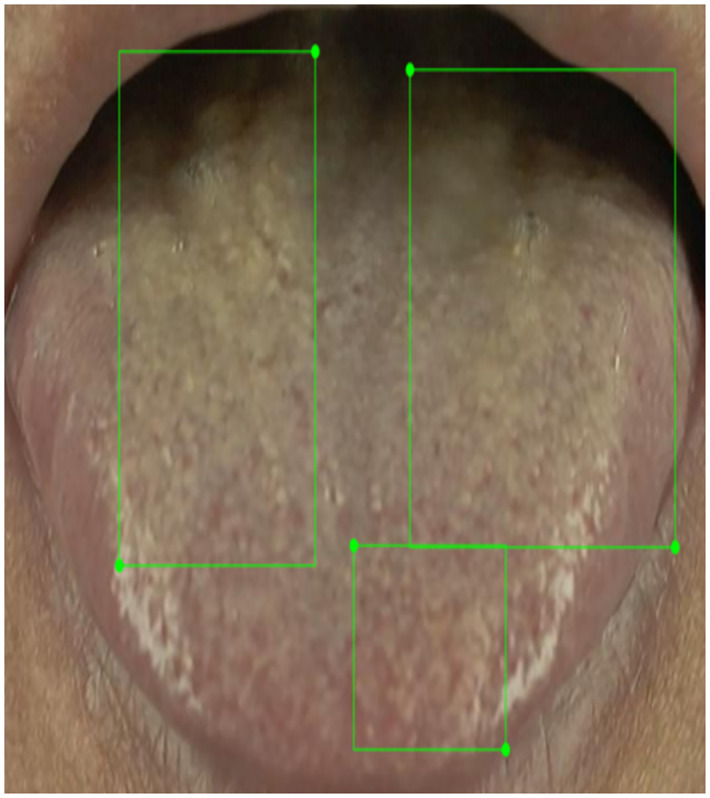
A tongue with multiple yellow coating areas.

Over two-thirds (69%) of tongue images have a yellow coating, and the uneven distribution of tongue coating can form multiple yellow coating areas, as shown in Fig 10. Almost all (98%) tongues with a yellow coating contain only one or two areas of yellow coating, as illustrated in Fig 5D.

### Distributions of area percentages of feature objects

**Fig 11:**
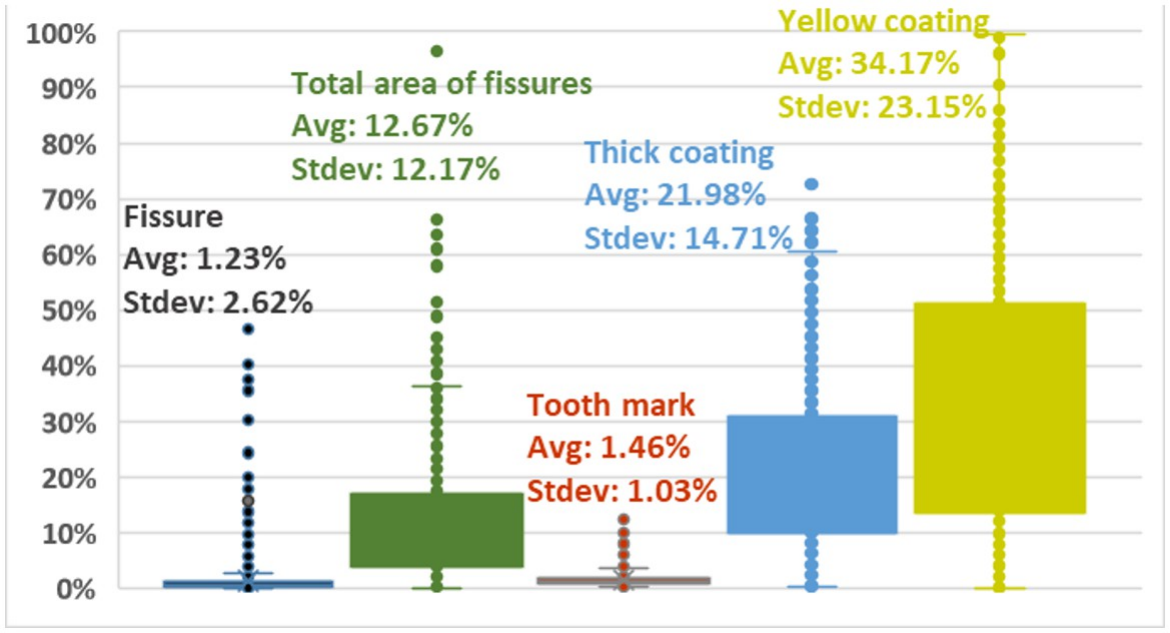
The distribution of area percentages of feature objects (with respect to the rectangle containing the whole tongue). Avg: average, Stdev: standard deviation.

Fig 11 illustrates the distribution of area percentages of feature objects (the rectangle containing the whole tongue is represented by a dashed green rectangle in Fig 2A). Marked differences exist regarding the numbers and areas of each tongue feature; the areas of yellow coatings, thick coatings and total areas of fissures are markedly larger than the areas covered by fissures and tooth marks. Notably, although the analysis identified a large total number of fissures and tooth marks, these objects are small and are therefore more difficult for AI model training to cope with than larger objects such as the areas of yellow coatings, thick coatings and total areas of fissures (see Table 1).

### Training results of the object detection model

The training results for each tongue feature are presented in Table 2 (Refer to S1 Table for details), which lists the best AP50 values, as well as training results for AP50 values with default settings and pretrained weights. At over 40 frames per second on a NVIDIA GeForce GTX 1060, the model was capable of detecting tongue features from any viewpoint in real time. The model size is only 22.4MB. Several models released after the completion of this study have also been trained (partially fine-tuned) for comparison (S2 Table). Among them PP-YOLOE+ [43] and DINO [44] show a more significant improvement in accuracy although the noticeable difference to the naked eye may not be significant. Nonetheless, the model sizes (773MB with PP-YOLOE+ and 839MB with DINO) are more than 34 times larger than the YOLOv4-tiny used in the original paper. The benefits of running this model on mobile devices require further evaluation.

**Table 2:** AP50 for tongue features.

	Fissure	Total area of fissures	Tooth mark	Thick coating	Yellow coating
AP50 after fine-tuned	47.67%	75.92%	58.94%	71.25%	59.78%
AP50 with default settings and pretrained weights	46.10%	61.18%	56.35%	61.18%	52.65%

In regard to the manual marking up of tongue features, fissures and tooth marks are less related to gradation and easier to annotate than thick coatings and yellow coatings, but the fissure and tooth mark areas are smaller and there are larger numbers of objects. AP50 values for the total areas of fissures and thick coatings both exceed 70%, while the AP50 values for tooth marks and yellow coatings are approximately 60% and the AP50 value for fissures is only 47.67%.

**Fig 12:**
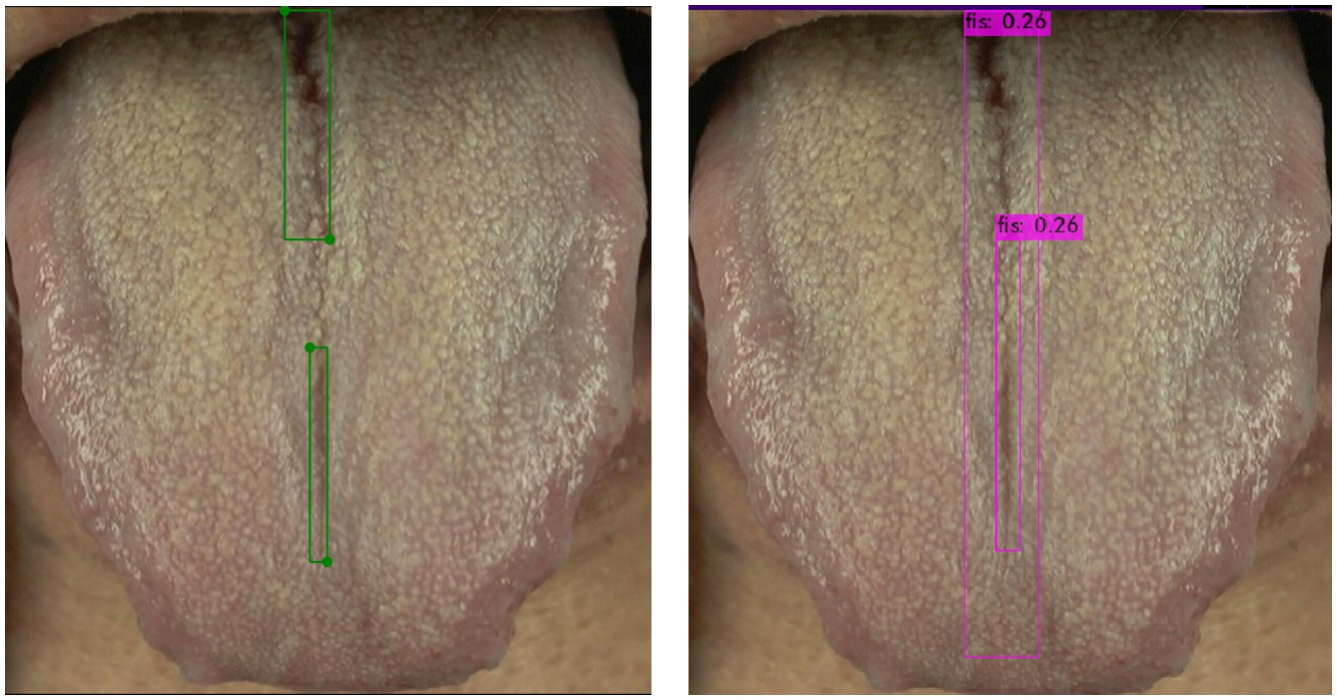
Multiple fissures are marked as a large fissure. The left is manually annotated, while the right is marked by the model.

The reason for the much higher AP50 value for the total area of fissures compared with the AP50 value for fissures may be due to the low input resolution (416*416) of the YOLOv4-tiny image used in this study, and because tongue fissures are not easily detected as they are mainly small objects. Twenty images were randomly selected from the test set for comparing the differences between expert manually annotated fissures and model-predicted fissures. Notably, ambiguities exist during manual interpretation; for instance, small tongue fissures may appear to be part of a larger fissure, which then yields lower AP50 values for fissures and therefore lower accuracy, as illustrated in Fig 12 and S8–S10 Figs. From the current results, the differences in the annotated areas between the model and manual annotations mostly stem from the acceptable ambiguous zones during interpretation and the predicted results provided by the model are satisfactory.

Training results for each setting (as presented in Table 3, and also in S1 and S2 Figs) are described as follows:

1. The accuracy of tongue fissure detection is not greatly affected by the inclusion or exclusion of pretrained weights. When pretrained weights are not used, the detection accuracies are higher for tooth marks, thick coatings and yellow coatings, but lower for fissures. Fissures are smaller than other features and are crisscrossed (as in Fig 6), making fissure detection difficult for AI models.
2. Color augmentation lowers the detection effect for thick coatings and yellow coatings, features more related to gradation than features such as tooth marks and fissures.
3. Reducing aspect ratios of images (*jitter*) decreases detection accuracy for all features, probably because the model loses the ability to identify features with varying aspect ratios. In general, increasing the varying degree of the network size (*network*) has positive benefits for each feature, whereas increasing the varying degree of image sizes (*resize*) results in obvious positive effects only for thick coating and yellow coating detection; the reason for this phenomenon is yet to be determined.
4. Compared with the model’s default learning rate of 0.00261, using another initial learning rate (0.005) or an adaptive learning rate policy (SGDR) yielded better results.

**Table 3:** Summary of training results of each setting.

Settings	Color augmentation		Jitter		Random		Resize		Learning rate		
Features	Enabled^a^^,^^b^	Disabled ^c^	1.3^a^	1.1	0^a^	1	1.5^a^	2	0.00261^a^	0.0005	SGDR
Fissure			V							V	V
Total area of fissures	V		V			V	V			V	V
Tooth mark	V		V			V	V			V	
Thick coating		V	V			V		V		V	V
Yellow coating		V	V			V		V			V

^a^ Default setting

^b^ Saturation = 1.5, Exposure = 1.5, Hue = 1;

^c^ Saturation = 1, Exposure = 1, Hue = 0

V: Recommended settings based on training experiments

A single model for simultaneously detecting all features were also trained. The training results are represented in S3 Fig. However, the mean AP50 (MAP50) value was only 50.98%; individual AP50s for fissures, tooth marks, thick coatings, yellow coatings, and total areas of features were 34.06%, 50.64%, 58.93%, 52.83%, and 58.46%, respectively. This may be because more than 80% of the objects were small objects such as fissures (21.76%) and tooth marks (61.82%) and also, the detection for each feature required different settings. Compared with the mean AP50 of YOLOv4-tiny on MS COCO 2017, which is about 42% [45], models trained in this study exhibits quite a good performance. Some detection results are represented in S4–S7 Figs.

YOLOv4-tiny has a very fast computing speed and is therefore particularly appropriate for mobile devices with limited computing power, such as smartphones or tablets. Trained models in this study can be used to record tongue features easily and dynamically under real-time conditions during the outpatient clinic visit. Users can take screenshots of the clearest viewing angle of tongue features for evidence, without needing to set up the shooting environment or wait for the results to be interpreted by TCM physicians.

Many studies are based on the development of YOLO, with various papers offering detailed reviews and comparisons [46, 47]. YOLO is famous for its speed, and there are still many other object detection models, each with its own strengths and weaknesses [48, 49]. Object detection models, including YOLO, are based on convolutional neural networks. Transformer-based models have recently entered the field of object detection and achieve similar accuracy [50]. However, due to their high computational demands, it remains challenging to deploy on mobile devices.

While deep learning has been applied to the analysis of tongue images, this AI modality is yet to be applied to the clinical interpretation of TCM tongue diagnosis. For the analysis of tongue images, the CHDNet model combines deep learning and support-vector machine classifiers to extract and classify tongue features [51]. However, the digital features extracted by the CHDNet model are not visual features and are therefore not related to tongue features quantified in TCM. Consequently, the digital features in the CHDNet model cannot be applied to clinical tongue inspection diagnosis. In addition, the classification results of this model show either “gastritis” or “no gastritis”, which does not relate to either a disease term or diagnosis in TCM language. In another deep learning model, the analysis of tongue color outperformed conventional imaging processing methods that lacked deep learning techniques [52]. Thus, these deep learning models do not relate to the positioning of tongue features and are therefore unsuitable for application in TCM clinical and teaching environments.

Up until now, only two studies exist in the literature that are similar to this study. In one study, deep learning object detection technology was used to mark tongue fissures and tooth marks with rectangles [13]. Notably, the marked rectangles in that study are much larger than the areas of tongue features, the MAP50 is only 34.42%, and the researchers do not describe details of the manually annotated dataset. The second study used the deep learning object detection technique, whereby rectangles marked tongue fissures, tooth marks, thick coatings, peeling coatings, and red dots [53]. However, these markings missed many obvious feature objects and the definition of accuracy was very rough: among many features on a single tongue, only one object with the best detection result was included in the final accuracy evaluation, instead of an evaluation of all detected objects.

This study has some limitations. First, the tongue feature dataset is smaller than other datasets used for object detection, and the dataset includes records from a single hospital, which may limit the diversity. Second, because the goal of this study is to apply tongue feature detection to mobile devices, the model is fast but compromises accuracy; a more accurate model can be used in high-end devices in future. Third, clinical verification is needed for the detection results of the model trained in this study. Clinical TCM physicians can provide feedback based on their domain knowledge and interactive analysis, all of which will improve the accuracy and practicality of the deep learning object detection model.

## Conclusions

This study has constructed a tongue feature dataset and trained a deep learning object detection model for locating tongue features that performs in real time. A step-by-step setting-tuning process were proposed to push the model to its limits as much as possible. Although there are differences in the features marked by manual and model annotations, these variances lie within the clinically acceptable gray area. As a result, the model’s predictions are deemed satisfactory. A real advantage of the training methods and materials used in this study is that they can be easily and quickly adopted in the training of any new model. In conclusion, we endorse the development of more AI applications that relate to tongue diagnosis in TCM, a traditional diagnostic technique that is informed by a huge body of expert experience accumulated over many centuries. Having more such AI applications will promote the wider use of this tool in the analysis of a patient’s overall health condition and also ensure that this technique continues to be valued in the future.

## Supporting information

S1 Fig Setting tuning results for fissures and total areas of fissure detection. (TIF)

S2 Fig Setting tuning results for tooth mark, thick coating and yellow coating detection. (TIF)

S3 Fig Setting tuning results for all tongue features detection. (TIF)

S4 Fig Fissures detected by our model. (TIF)

S5 Fig Tooth marks detected by our model. (TIF)

S6 Fig Thick coatings detected by our model. (TIF)

S7 Fig Yellow coatings detected by our model. (TIF)

S8 Fig Correct fissure detection. (TIF)

S9 Fig Good fissure detection. (TIF)

S10 Fig Acceptable fissure detection. (TIF)

S1 Table Evaluation metrics for tongue features. (DOCX)

S2 Table Comparison of models for tongue features. (DOCX)
