# Supplementary figures and images for: Tongue feature dataset construction and real-time detection
Source: PLoS One. 2024 Mar 7;19(3):e0296070. doi: 10.1371/journal.pone.0296070 (PMC10919637; doi:10.1371/journal.pone.0296070)

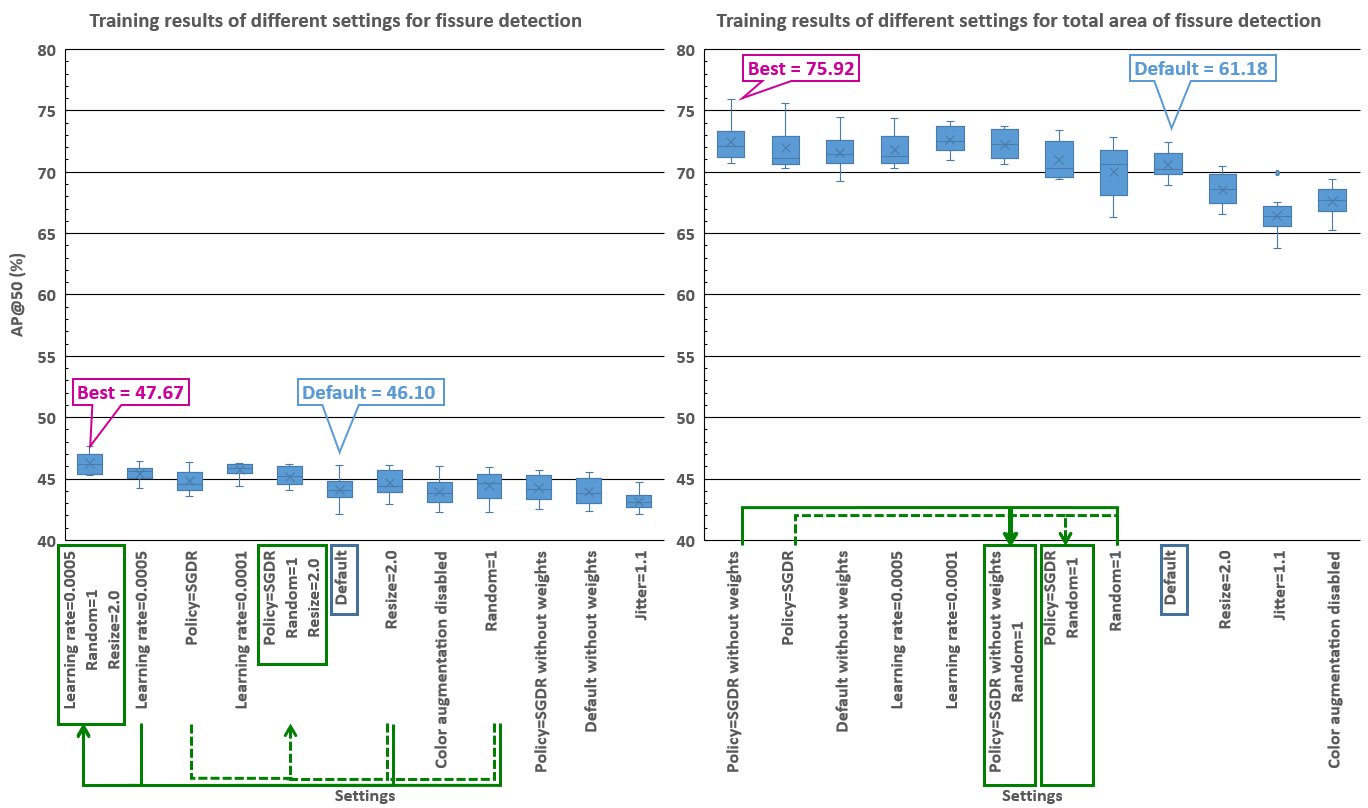

Supplement: S1 Fig — (TIF) [file pone.0296070.s001.tif]

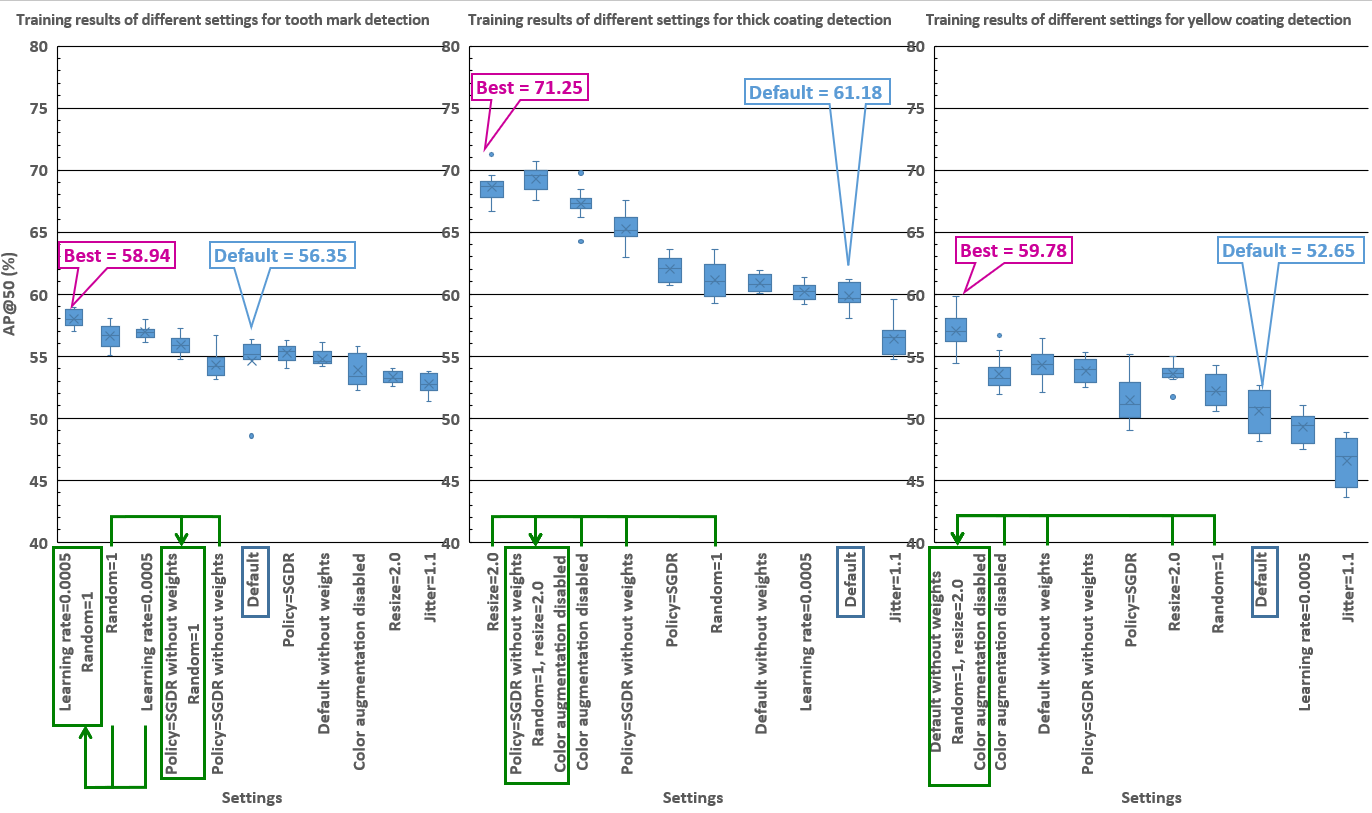

Supplement: S2 Fig — (TIF) [file pone.0296070.s002.tif]

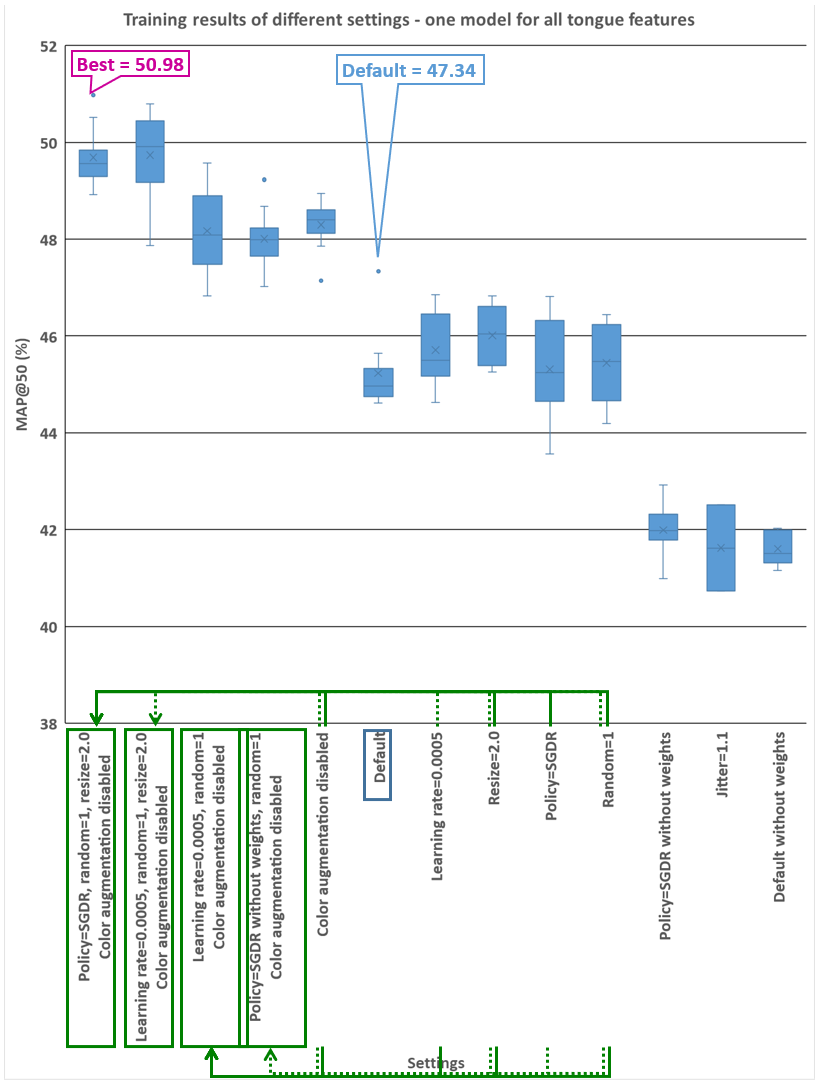

Supplement: S3 Fig — (TIF) [file pone.0296070.s003.tif]

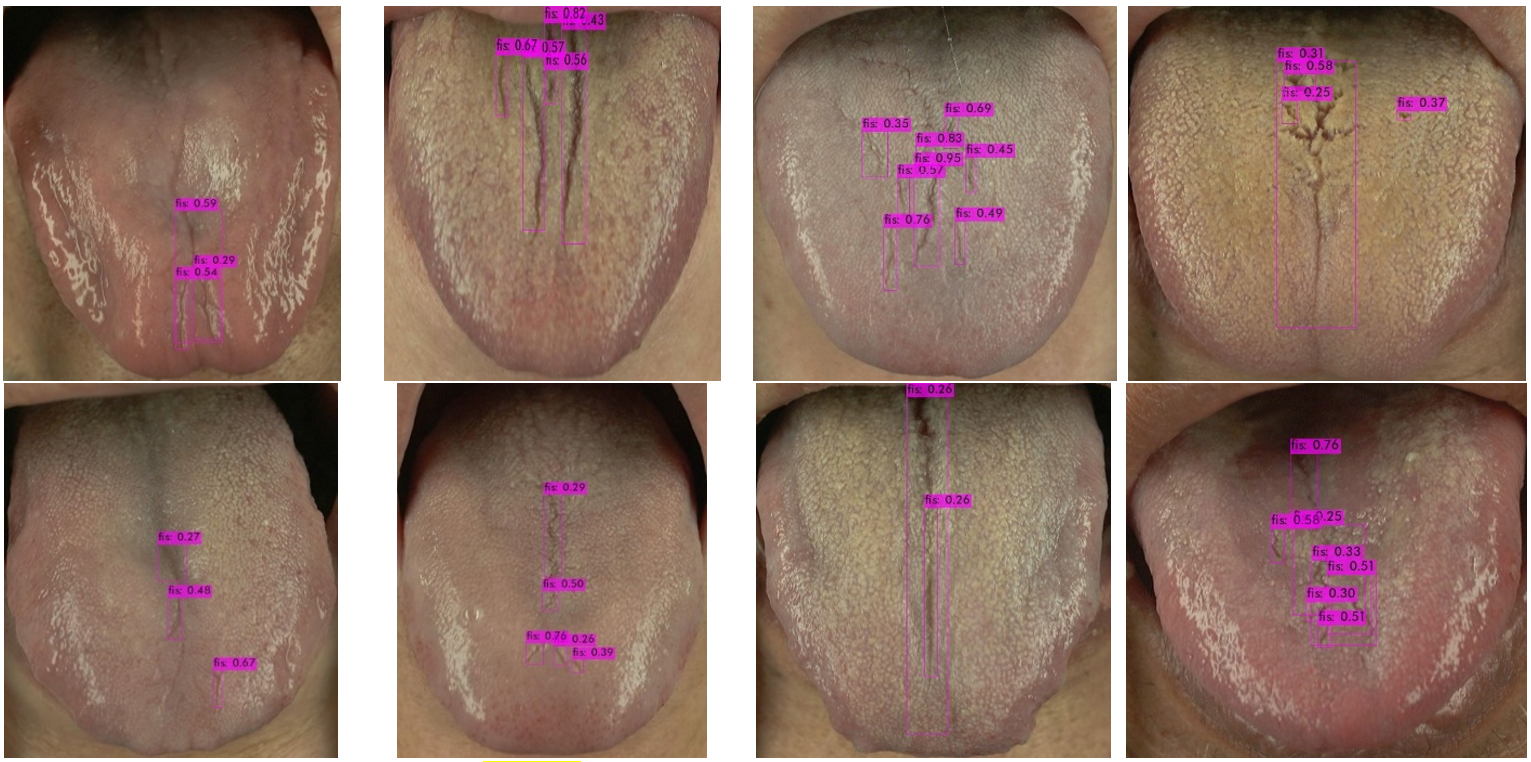

Supplement: S4 Fig — (TIF) [file pone.0296070.s004.tif]

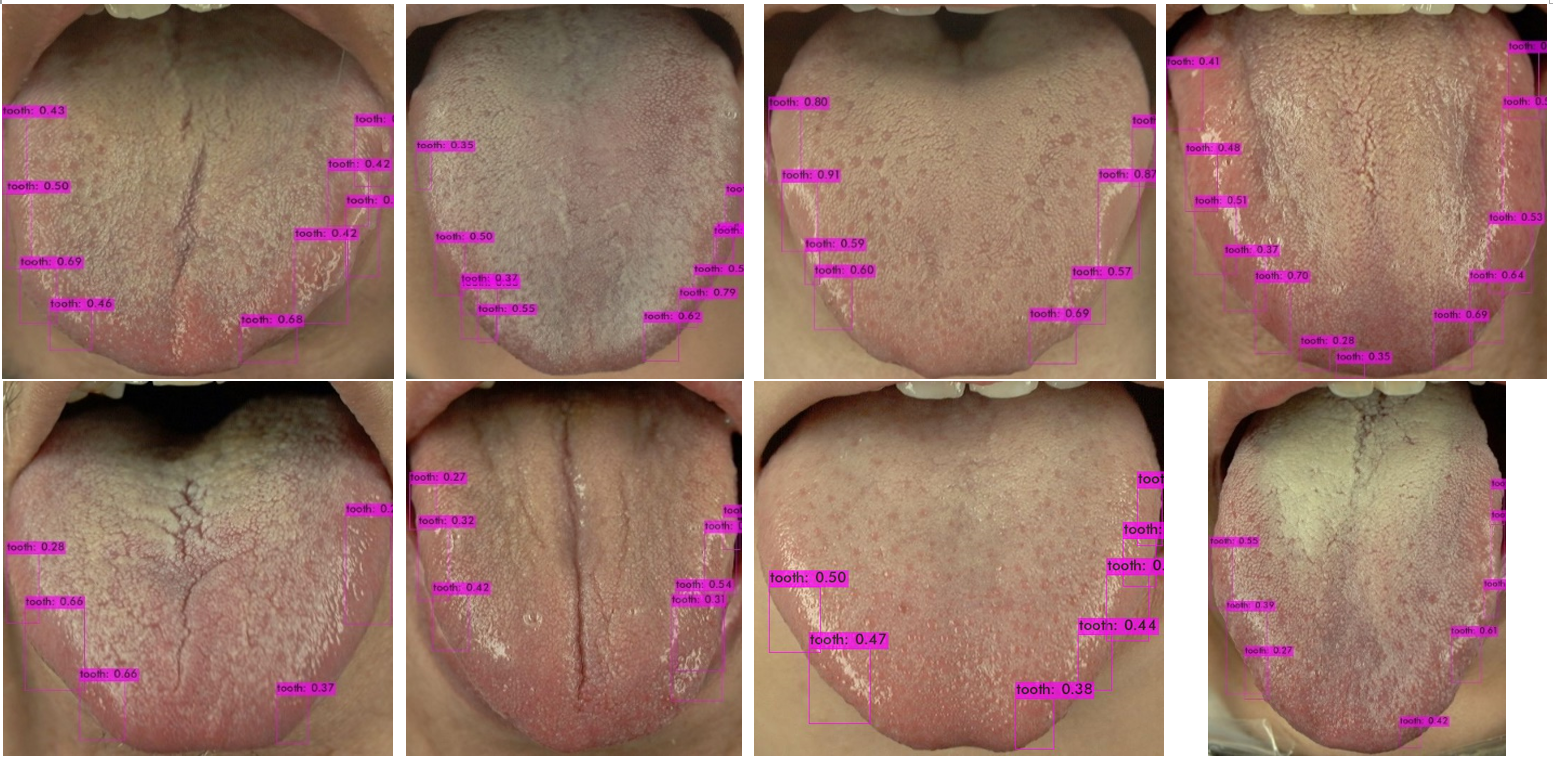

Supplement: S5 Fig — (TIF) [file pone.0296070.s005.tif]

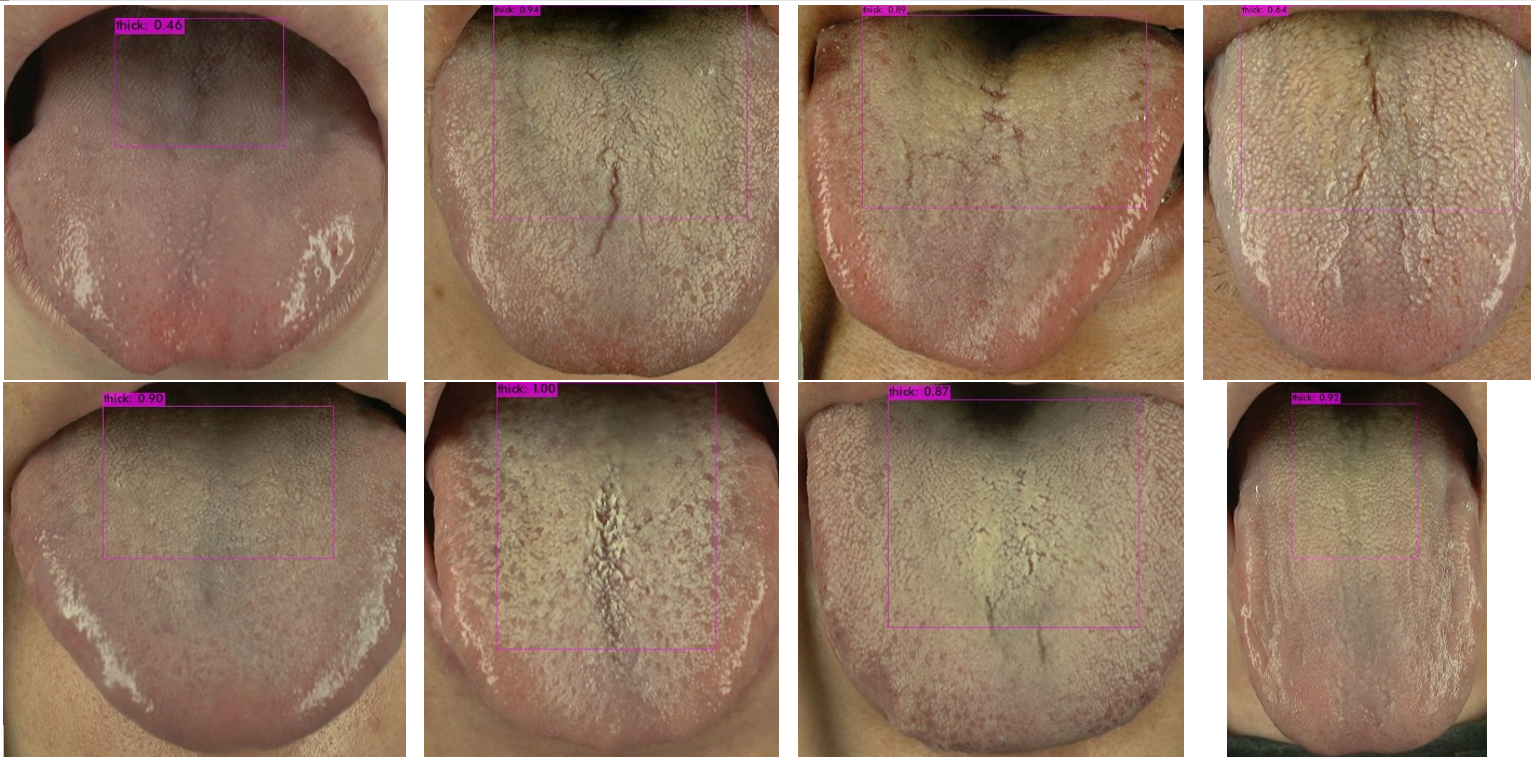

Supplement: S6 Fig — (TIF) [file pone.0296070.s006.tif]

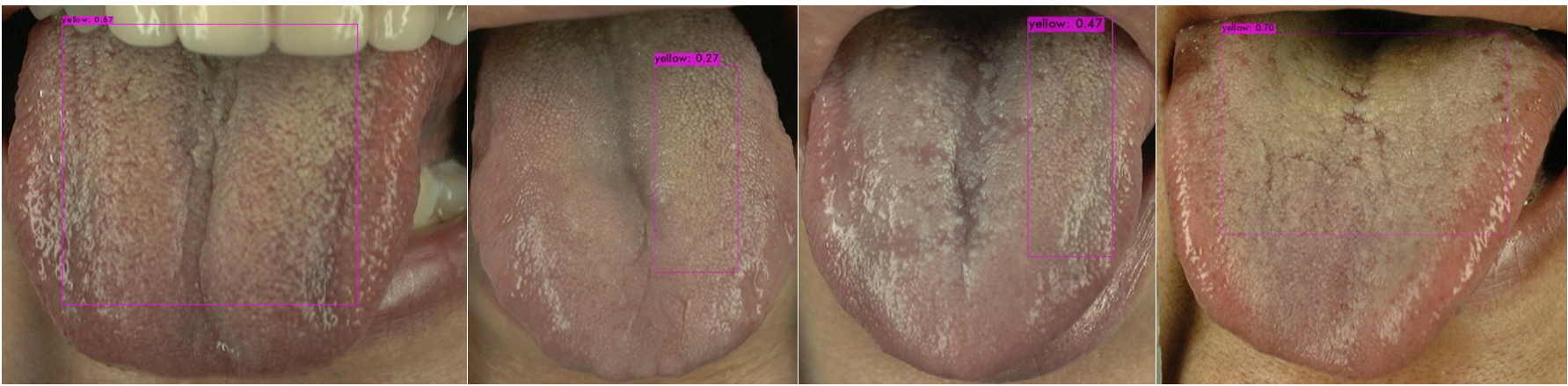

Supplement: S7 Fig — (TIF) [file pone.0296070.s007.tif]

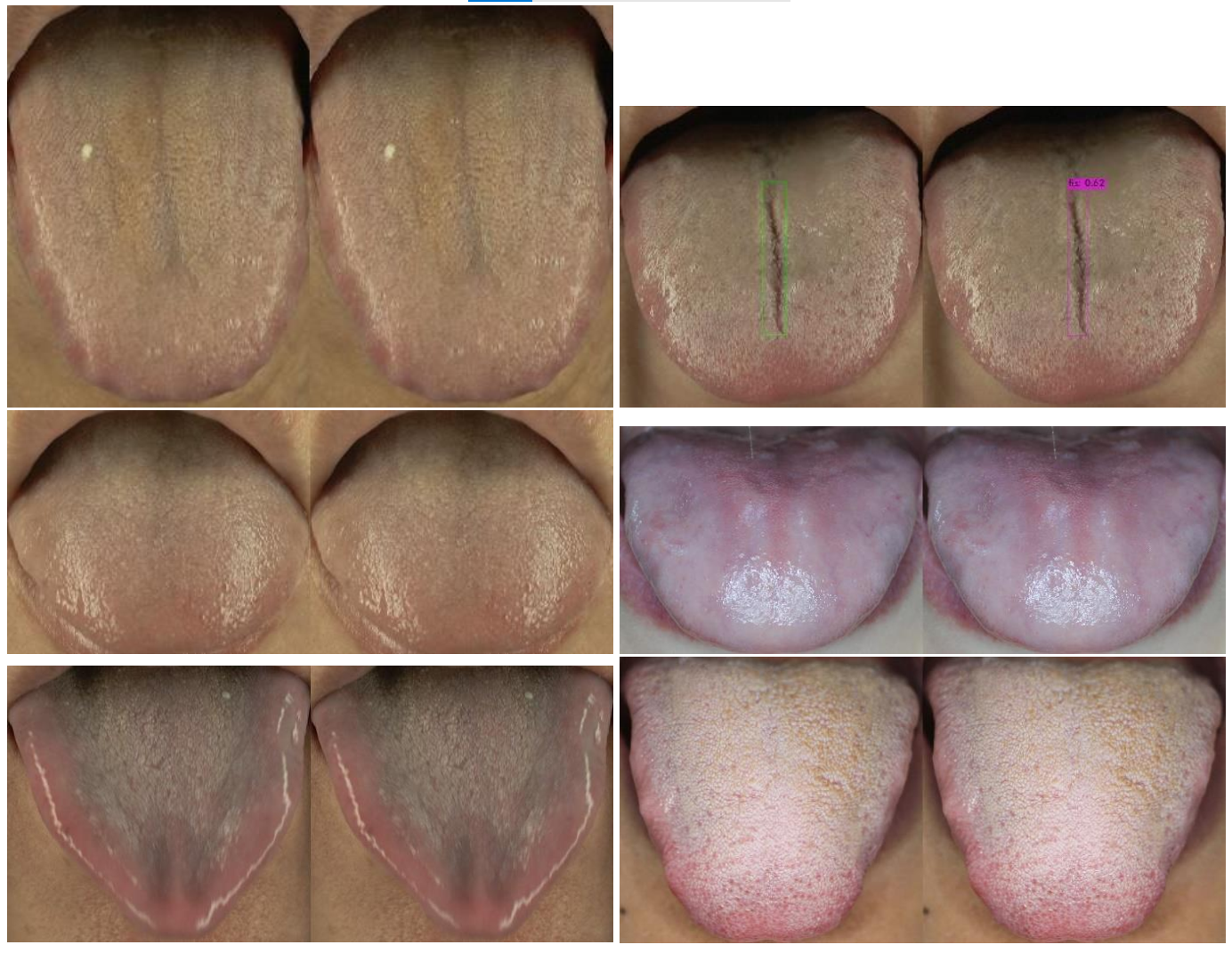

Supplement: S8 Fig — (TIF) [file pone.0296070.s008.tif]

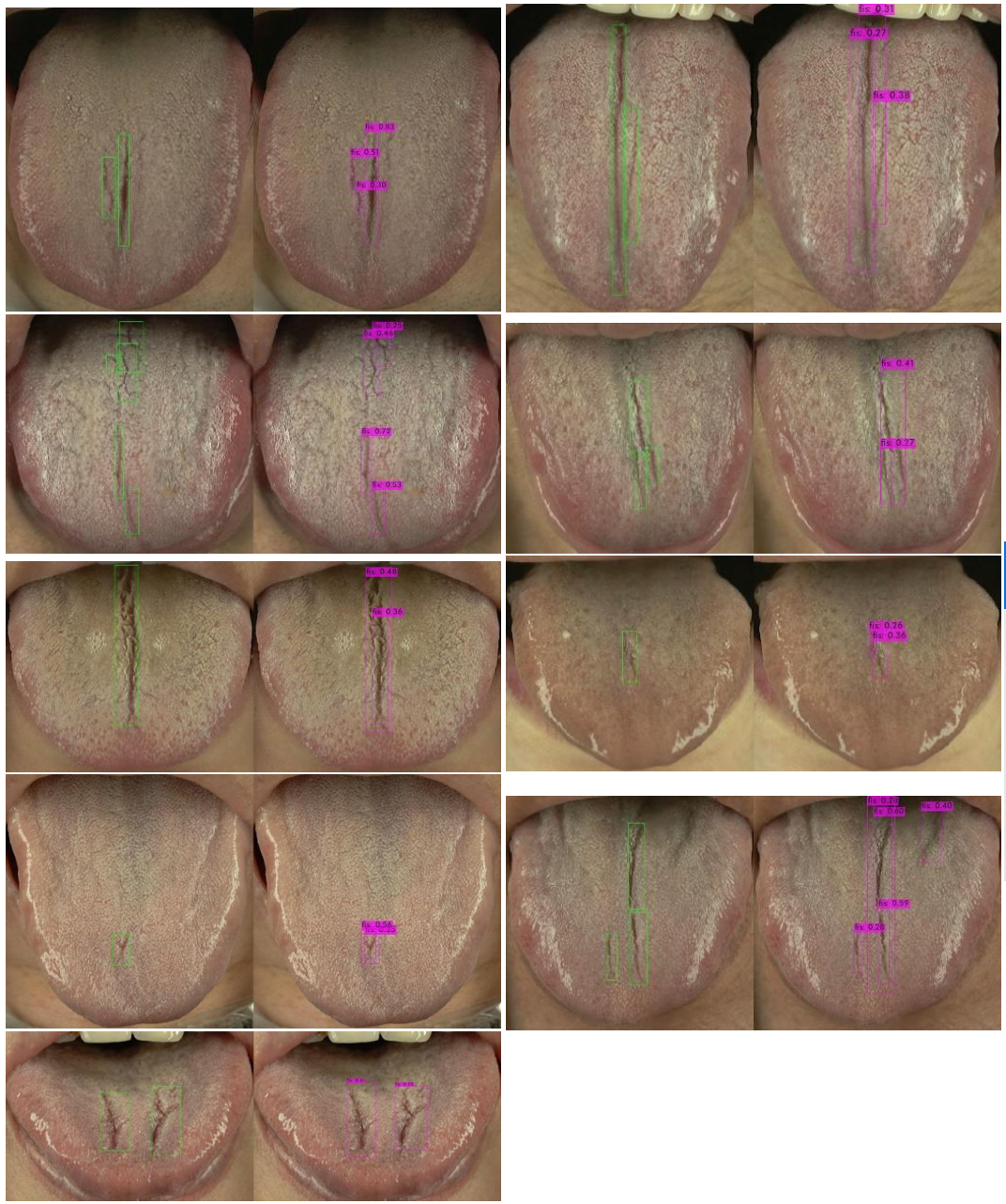

Supplement: S9 Fig — (TIF) [file pone.0296070.s009.tif]

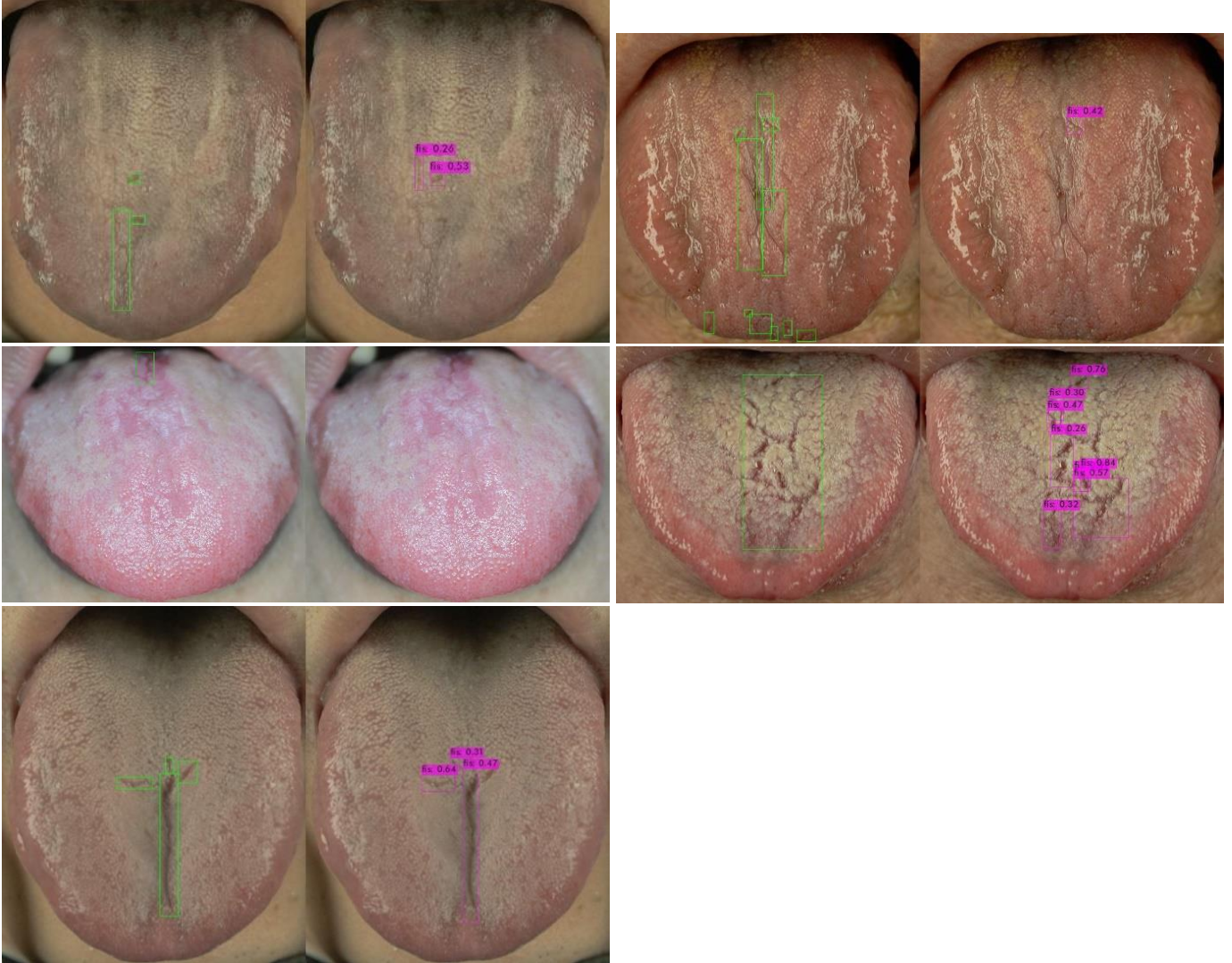

Supplement: S10 Fig — (TIF) [file pone.0296070.s010.tif]
