# Supplementary material for: Tongue feature dataset construction and real-time detection
Source: PLoS One. 2024 Mar 7;19(3):e0296070. doi: 10.1371/journal.pone.0296070 (PMC10919637; doi:10.1371/journal.pone.0296070)
Supplement: S1 Table — (DOCX) [file pone.0296070.s011.docx]

**S1 Table. Evaluation metrics for tongue features**

|  | Precision | Recall | F1-score | TP | FP | FN | IoU | AP50 |
| --- | --- | --- | --- | --- | --- | --- | --- | --- |
| Fissure | 0.58 | 0.44 | 0.5 | 294 | 212 | 368 | 40.67 | 47.67 |
| Total area of fissures | 0.83 | 0.66 | 0.74 | 100 | 20 | 51 | 57.1 | 75.92 |
| Tooth mark | 0.64 | 0.54 | 0.59 | 1228 | 680 | 1037 | 44.8 | 58.94 |
| Thick coating | 0.85 | 0.56 | 0.68 | 149 | 27 | 116 | 60.63 | 71.25 |
| Yellow coating | 0.77 | 0.43 | 0.55 | 75 | 22 | 100 | 55.36 | 59.78 |

F1-score = (precision * recall) / (precision + recall)
TP: True positive
FP: False positive
FN: False negative
IoU: Intersection over union
AP50: average precision with an intersection over union greater than or equal to 50%
