# Supplementary material for: Tongue feature dataset construction and real-time detection
Source: PLoS One. 2024 Mar 7;19(3):e0296070. doi: 10.1371/journal.pone.0296070 (PMC10919637; doi:10.1371/journal.pone.0296070)
Supplement: S2 Table — (DOCX) [file pone.0296070.s012.docx]

**S2 Table. Comparison of models for tongue features**

| Model information | | Tongue features | | | | |
| --- | --- | --- | --- | --- | --- | --- |
| Model name | Model size | Fissure | Total area of fissures | Tooth mark | Thick coating | Yellow coating |
| YOLOv4-tiny (default settings)^a^ | 22.0 MB | 46.1% | 61.2% | 56.4% | 61.2% | 52.7% |
| YOLOv4-tiny (fully fine-tuned)^a^ | 22.0 MB | 47.7% | 75.9% | 58.9% | 71.3% | 59.8% |
| YOLOv5n^b^ | 3.9 MB | 38.1% | 75.5% | 60.8% | 77.8% | 57.7% |
| YOLOv5s^b^ | 14.1 MB | 37.7% | 71.8% | 60.6% | 75.1% | 58.6% |
| YOLOv7-tiny^c^ | 46.4 MB | 41.3% | 59.3% | 57.1% | 76.9% | 55.7% |
| YOLOv7-w^c^ | 619.0 MB | 48.5% | 75.8% | 60.2% | 76.7% | 58.5% |
| YOLOv8n^b^ | 6.2 MB | 50.8% | 78.7% | 58.6% | 76.4% | 60.0% |
| YOLOv8x^b^ | 130.0 MB | 49.9% | 74.9% | 56.8% | 76.8% | 60.4% |
| PP-YOLOE+^c^ | 773.0 MB | 55.5% | 80.4% | 61.7% | 75.0% | 60.8% |
| DINO^d^ | 839.0 MB | 61.0% | 84.7% | 66.4% | 77.2% | 61.2% |

^a^ YOLOv4 source: <https://github.com/AlexeyAB/darknet>

^b^ YOLOv5 and YOLOv8 sources (the newest version): <https://github.com/ultralytics/>. YOLOv5 and YOLOv8 have undergone multiple versions, but neither has been accompanied by a published paper.

^c^ YOLOv7 and PP-YOLOE+ sources: <https://github.com/open-mmlab/mmyolo>

^d^ DINO source: <https://github.com/open-mmlab/mmdetection>
